# Multisystem inflammatory syndrome in children and Kawasaki disease

**DOI:** 10.3389/fimmu.2025.1554787

**Published:** 2025-04-15

**Authors:** Ancuta Lupu, Cristina Gavrilovici, Cristina Maria Mihai, Denisa Claudia Tonco, Alin Horatiu Nedelcu, Leonard Pertea, Tatiana Chisnoiu, Ginel Baciu, Ramona Mihaela Stoicescu, Delia Lidia Salaru, Minerva Codruta Badescu, Magdalena Cuciureanu, Olga Cirstea, Vasile Valeriu Lupu

**Affiliations:** ^1^ Pediatrics, Faculty of Medicine, “Grigore T. Popa” University of Medicine and Pharmacy, Iasi, Romania; ^2^ Faculty of Medicine, “Ovidius” University, Constanta, Romania; ^3^ Faculty of Medicine, “Grigore T. Popa” University of Medicine and Pharmacy, Iasi, Romania; ^4^ Pediatrics, Faculty of Medicine and Pharmacy, “Dunarea de Jos” University of Galati, Galati, Romania; ^5^ Pediatrics, “Nicolae Testemitanu” State University of Medicine and Pharmacy, Chisinau, Moldova

**Keywords:** multisystem inflammatory syndrome in children, Kawasaki disease, SARS-CoV-2, immune response, inflammatory response, pediatrics

## Abstract

This narrative review aims to analyze and compare the current literature on multisystem inflammatory syndrome in children (MIS-C) and Kawasaki disease (KD), with a focus on case definitions, clinical features, diagnostic approaches, treatment strategies, and outcomes. Through a comprehensive review of relevant studies, including screening titles, abstracts, and full-text articles, key similarities and differences were identified. Both MIS-C and KD involve immune system dysregulation and share clinical manifestations such as rash, gastrointestinal symptoms, and cardiovascular involvement, with treatments often centered around immunomodulatory therapies. However, significant differences were observed, particularly in terms of age distribution, demographic prevalence, clinical presentation, and diagnostic criteria, with KD primarily affecting younger children and being associated more prominently with coronary artery abnormalities. While both diseases raise concerns about severe cardiac involvement and the need for intensive care, their pathogenic mechanisms have not been fully understood. Ongoing research is critical to elucidating these mechanisms, refining diagnostic criteria, and optimizing therapeutic approaches to improve outcomes for affected children. This comparative analysis is essential for advancing the understanding of both conditions, as accurately distinguishing between MIS-C and KD has significant implications for clinical decision-making and patient management. Given their overlapping yet distinct clinical features, precise differentiation is critical for ensuring timely diagnosis, optimizing therapeutic strategies, and improving patient outcomes. The concern among pediatric patients stems from the potential for severe complications, particularly cardiac involvement, which underscores the need for heightened awareness, early recognition, and evidence-based treatment strategies to minimize long-term morbidity and mortality.

## 1 Introduction

Multisystem inflammatory syndrome in children (MIS-C) is a hyperinflammatory condition that affects children and adolescents 2–6 weeks after SARS-CoV-2 infection. It primarily affects children aged 6–12 years and shows a predominance in male individuals (1). The first cases of this syndrome were reported in the United Kingdom, Italy, and the United States. Verdoni and colleagues first described it in 2020, detailing seven boys and three girls aged 5–7 years in Bergamo, Italy, who were suffering from a Kawasaki-like disease (2). This condition had a higher incidence from February to April 2020 than Kawasaki disease (KD) registered in the previous 5 years (3). In 2019, following the spread of coronavirus globally, there were reported increases in cases of children presenting with a Kawasaki-like disease that could also be accompanied by prolonged fever, rash, and conjunctivitis (4).

The first case definition was issued by the Centers for Disease Control and Prevention (CDC) in 2020. Using this case definition, the incidence in the United States was estimated at 5.1 cases per million people per month from April to June. Most of the cases were among non-Hispanic Black, Hispanic, or non-Hispanic Asians compared to non-Hispanic white individuals (5). In 2022, the CDC and the Council of State and Territorial Epidemiologists (CSTE) created a new surveillance case definition that went into effect on January 1, 2023. Approximately 87% of cases reported met the 2023 case definition (6). The condition was initially termed pediatric inflammatory multisystem syndrome temporally associated with SARS-CoV-2 (PIMS-TS) (5, 7). This definition was formulated by experts in the United Kingdom and published by the Royal College of Paediatrics and Child Health, with a corresponding article in *The Lancet* on May 7, 2020. The publication detailed nine children in south London with PIMS-TS requiring critical care, highlighting the severe nature of the disease. Prior to this, it was known that children infected with SARS-CoV-2 typically experienced minimal symptoms (8). The emergence of this novel syndrome has raised significant concern among healthcare professionals and parents, as it shares clinical features with toxic shock syndrome (TSS), secondary hemophagocytic lymphohistiocytosis (SHLH), macrophage activation syndrome (MAS), and KD. KD, also called mucocutaneous lymph node syndrome, is an acute and usually self-limiting vasculitis of the small and medium caliber arteries, which almost exclusively affects children below 5 years of age (9, 10). It is a leading cause of acquired heart disease in children (11). This condition is characterized by an acute, self-limiting vasculitis that particularly targets the coronary arteries in previously healthy young infants and children. Although it has been over 50 years since Dr. Tomisaku Kawasaki first identified this disease in Japan in 1967, its exact cause remains unclear. The prevailing hypothesis suggests that it may stem from an abnormal immune response to one or more unknown pathogens in genetically predisposed individuals. In the acute phase of the disease, patients with KD may have a condition known as Kawasaki disease shock syndrome (KDSS), which includes hemodynamic instability. Other patients with KD may fulfill the criteria of MAS, resembling secondary hemophagocytic lymphohistiocytosis (12).

We conducted an extensive narrative review comparing MIS-C and KD, analyzing data from clinical studies and reviews. Completed over a 6-month period, this review encompasses various aspects of both conditions, from etiology to treatment methods. We searched major international databases for relevant articles using the keywords “MIS-C and Kawasaki disease”, “Kawasaki-like disease”, and “multisystemic inflammatory syndrome due to SARS-CoV-2” combined with “clinical comparison” and “pediatric”. Furthermore, we conducted a manual review of eligible original articles by assessing the references from the initial search results, reviews, and other relevant literature.

## 2 Etiology and epidemiology

MIS-C is a condition that appears 2–6 weeks after the SARS-CoV-2 infection and affects children aged 6–12 years, with a prevalence for the male gender. In 2023, the median age for MIS-C was 7 years, a shift to a younger age group compared to that in 2022 when the median age was reported to be 9 years. Race may be a potential risk factor, as a higher incidence of MIS-C has been observed in children of African, Hispanic, or South Asian origin (13). Studies from Japan and South Korea have reported a lower incidence of MIS-C, as well as fewer cases requiring intensive care and no deaths. Of these cases, 80% were encountered during the Omicron period (14, 15). The CDC from the United States reports that the incidence is declining from a peak of 6.79 cases per million persons in 2021 to 0.11 cases per million persons in 2023, with a high incidence during the fall when the overall number of COVID-19 cases was rising (6). In 2023, 80% of the US-reported cases were vaccine-eligible but were unvaccinated children (16). Many children with MIS-C have had fewer or no symptoms of COVID-19. In the pediatric population, the risk of severe COVID-19 was elevated in neonates, preterm infants, and children with underlying health conditions (17). Among these, obesity was the most commonly encountered comorbidity (18), along with diabetes, heart and neurological diseases, chronic lung conditions, asthma, and compromised immune systems (17). Recent findings suggest that SARS-CoV-2 infection may trigger the onset of type 1 and type 2 diabetes in children, which is a common chronic metabolic disorder in this age group. Some studies have shown that in patients with SARS-CoV-2 infection, those with diabetes had a higher likelihood of hospitalization. Furthermore, the data indicated that diabetes and other cardiovascular conditions are significant risk factors for adverse outcomes and mortality in COVID-19 patients (19, 20). The lower expression of the angiotensin-converting enzyme (ACE)-2 receptor gene, which is the target of SARS-CoV-2, may partly explain why children tend to experience milder COVID-19 symptoms compared to adults. This, along with fewer comorbidities and a trained innate immune system, likely contributes to their reduced disease severity (21). While multisystemic inflammatory syndrome is linked to COVID-19, the cause of KD remains unknown (22, 23). Experts suggest that a prior viral infection may be responsible. This self-limiting condition shows higher prevalence during winter and spring. Occurring more frequently in male individuals, KD mainly affects children under 5 years old, with the highest incidence between 18 and 24 months, particularly in predominantly White communities (3, 22, 23). Female individuals are affected approximately one-third as often as male individuals, and infants younger than 4 months are rarely impacted. The condition is most prevalent among children of Japanese and Asian descent compared to other regions globally (24). In East Asian countries, MIS-C remains relatively rare, whereas KD has the highest incidence globally (14). Studies from Japan have also indicated a higher occurrence in children with a family history of KD compared to the general population (8). While the etiology remains unknown, many case reports have linked KD with many viral agents like cytomegalovirus, adenovirus, rhinovirus, enterovirus, and bocavirus (25, 26). However, there are studies that have compared the X-ray findings in *Mycoplasma pneumoniae* infection with other types of pneumonia developed in KD, which show results that dispute the viral etiology theory (10, 23). While some studies have suggested a possible link between KD and environmental factors such as fungal toxins and pollution (27), no research has definitively confirmed this association (28–30).

## 3 Immune response and physiology

Studies of both KD and MIS-C have shown increased interleukin (IL)-1, IL-6, and IL-18 secretion as well as signaling in tumor necrosis factor (TNF) and interferon-gamma (IFN-γ) pathways that cause the release of various cytokines that can further lead to cytokine storms (31, 32).

**Figure 1:**
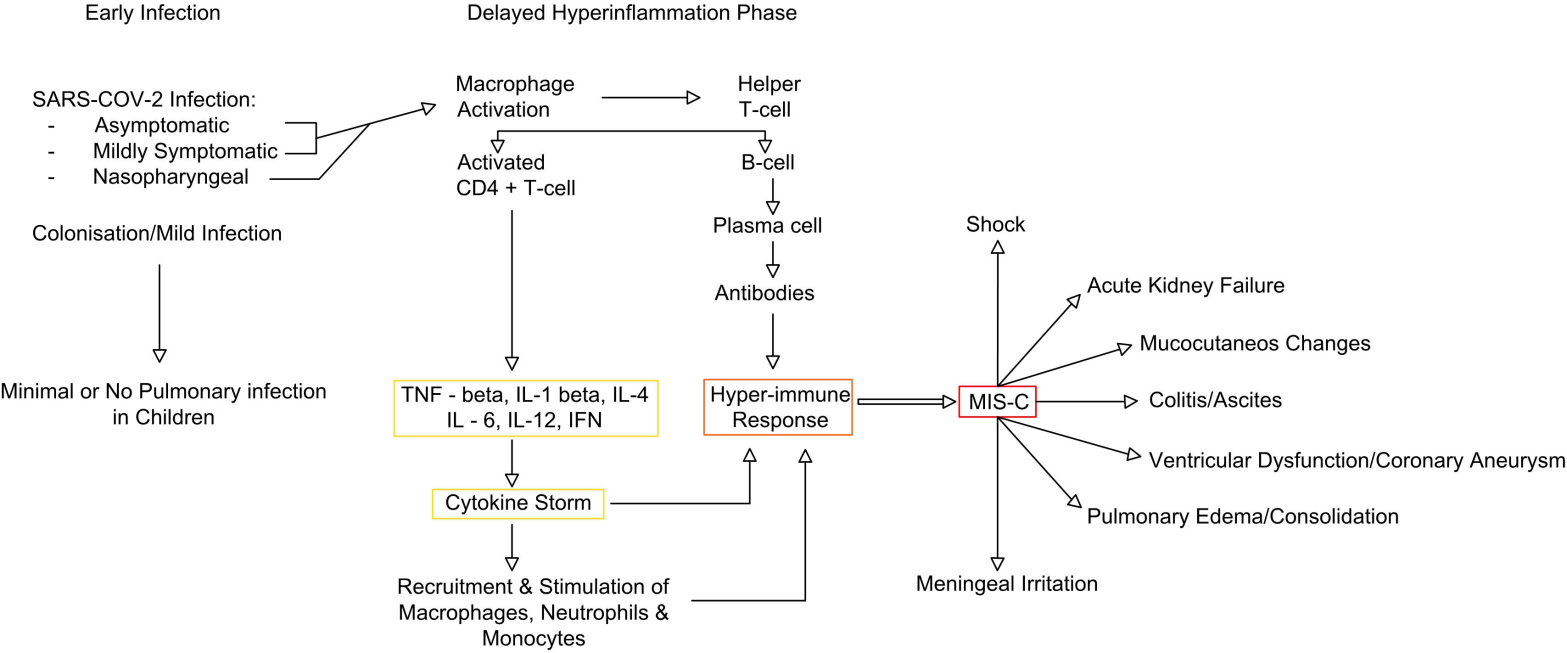
MIS-C immune pathway (adapted from 35). MIS-C, multisystem inflammatory syndrome in children.

In MIS-C, residual COVID-19 viral particles persist in infected tissues, including myocardial cells, even after the virus has been cleared from the body. Viral RNA was detected in 35% of myocardial infarctions in individuals with another SARS-CoV. The direct viral impact is attributed to the interaction between the virus and ACE-2 receptors, which are found on the surface of endothelial and endocardial cells (21). These viral particles trigger a form of molecular mimicry, causing the immune system to mount an exaggerated inflammatory response (32) (**Figure 1**). The activation of monocytes and macrophages leads to a predominance of neutrophils. Carter et al. reported increased neutrophil and monocyte activation in the acute phase of MIS-C, indicated by high CD64 expression. This aligns with the findings of upregulated CD54 and CD64 in these cells. Additionally, antigen-presenting cells, including monocytes, dendritic cells, and B cells, exhibit low CD86 and HLA-DR expression, suggesting impaired antigen presentation. Monocyte profiling may help distinguish MIS-C from severe COVID-19, as patrolling monocytes dominate in MIS-C, whereas HLA-DRlo classical monocytes are more prevalent in severe COVID-19 (33). Complement activation, likely through the lectin pathway, has been observed in some MIS-C patients, as evidenced by elevated soluble C5b-9 levels. In MIS-C, CD8+ T-cell counts are significantly lower compared to those in children with mild SARS-CoV-2 infection. However, many of the remaining T cells exhibit activation markers, such as CD38 and HLA-DR. This is the main difference between MIS-C and TSS, which is typically triggered by bacterial superantigens like staphylococcal enterotoxin B and streptococcal mitogenic exotoxin Z. Although clinically they share similarities, in TSS, superantigen exposure leads to widespread T-cell activation and excessive proinflammatory cytokine release, resulting in multiorgan damage—paralleling the immune dysregulation seen in MIS-C (34). Regarding the B-cell response, patients with MIS-C exhibit reduced levels of total, effector, and memory B cells compared to healthy individuals, along with an increase in circulating plasmablasts. These short-lived plasmablasts may produce autoantibodies targeting self-antigens. Also, Gruber et al. identified anti-La autoantibodies in MIS-C patients, a finding commonly associated with systemic lupus erythematosus (SLE) and Sjogren’s disease. These findings suggest a potential autoimmune component in MIS-C pathogenesis (34, 35). The resulting inflammation and cytokine release cause cardiac damage as the immune system tries to eliminate the remaining viral particles. The exaggerated T-cell response to the SARS-CoV-2 spike glycoprotein leads to the formation of autoantibodies against cardiovascular, gastrointestinal, and endothelial antigens (33). The activation of IFN-γ increases human leukocyte antigen presentation in tissues, creating a more “sensitized” immune response. This, along with the activation of the IL-1 and IFN-γ pathways, triggers a response from CD8 cytotoxic T cells. These T cells migrate to the heart and other organs, damaging the tissues where the virus is present. Studies have shown that total T-cell frequencies were lower in both MIS-C and KD compared to healthy children, but MIS-C has a higher subpopulation of central memory and effector memory CD4+ T cells and a lower subpopulation of naïve CD4+ T cells when compared to KD. The levels of follicular helper T cells, which support B-cell responses, and CD8+ T cells were decreased in patients with multisystem inflammatory syndrome (36).

KD is an acute vasculitis that affects medium-sized arteries including the coronary arteries (35). Although vascular inflammation is most pronounced in the coronary vessels, vasculitis can also occur in veins, capillaries, small arterioles, and larger arteries (37, 38). The underlying pathogenic mechanism is unknown. An exaggerated immune reaction is implicated, including both innate and adaptive systems, following a possible viral infection (39). Immune complexes are formed, which signal the proliferation of monocytes and macrophages, resulting in neutrophilia, a CD8+ lymphocyte proliferation, and an increased level of immunoglobulin A (IgA)-producing plasma cells. Apart from the immune complex reactions, IgA plays a role in KD, and studies have suggested that elevated IgA levels correlate with coronary artery involvement (40). Current research indicates that patients with MIS-C exhibit elevated levels of IgA and immunoglobulin G (IgG). The IgA elevated levels are linked with the immune responses of the gastrointestinal tract. Children with MIS-C develop a stronger IgA response in comparison to children with COVID-19, while IgG and immunoglobulin M responses are similar in both groups. The presence of several IgG and IgA autoantigens—some of which are associated with autoimmune diseases like SLE, Sjogren’s disease, polymyositis, and dermatomyositis—were also reported (41). A link cannot yet be established between lower or higher levels of IgA and the severity or frequency of complications in this pathology at pediatric age (42, 43).

Several proteins are involved in the pathogenesis of KD; N-terminal prohormone of brain natriuretic peptide (BNP) can be a potential biomarker for myocardial injury. Tenascin-C, an extracellular glycoprotein, can be used as a predictor of coronary artery aneurysm (CAA) development (44, 45). The inflammatory cells secrete various cytokines, ILs, and matrix metalloproteinases, which lead to the damage of internal elastic lamina and necrosis of smooth muscle cells. IL-1 appears to be the key factor in the pathogenesis of the disease. During the acute phase, high concentrations of IL-1 have been identified in the peripheral blood. The severe inflammation caused by immune complexes triggers a cytokine release that results in organ injury (46). High concentrations of TNF-α had also been identified in the peripheral blood of patients diagnosed in the acute phase (44).

As a conclusion to this subchapter, MIS-C and KD exhibit distinct immunological profiles despite some overlapping features. MIS-C is thought to involve a superantigen-driven mechanism, whereas KD likely results from an immune response to a conventional antigen (47). Additionally, MIS-C is characterized by differences in T-cell subsets, including lower levels of naïve CD4+ and follicular helper T cells but higher levels of central and effector memory CD4+ T cells compared to those in KD. Whether these syndromes are distinct or part of a shared immunopathogenic spectrum remains under investigation (33).

## 4 Clinical comparison

Multisystem clinical findings common to MIS-C and KD—such as rash, fever, gastrointestinal tract abnormalities, swollen lymph nodes, headaches, and fatigue—also overlap with clinical manifestations triggered by other pediatric viral illnesses (48).

For patients diagnosed with multisystemic inflammatory syndrome, the most common symptom is a fever that lasts for more than 24 hours. It is usually associated with at least one of the following: abdominal pain, vomiting, diarrhea, skin rash, and mucocutaneous lesions (i.e., conjunctivitis). In severe cases, children can present with hypotension and shock (49). The signs and symptoms of MIS-C vary among children, and some may exhibit symptoms not mentioned here. MIS-C can develop weeks after a child has been infected with SARS-CoV-2, even if the infection was asymptomatic or unknown to the child and caregivers. Gastrointestinal inflammation in MIS-C can present with abdominal pain, vomiting, and diarrhea, sometimes leading to a misdiagnosis of acute appendicitis. Neck pain is also reported and may be accompanied by phlegmon visible on radiographic imaging. Neurologic involvement in children with MIS-C is usually transient, manifesting as headaches, myalgia, or fatigue (50, 51). Although rare, severe neurologic symptoms can include encephalopathy, stroke, demyelination, fulminant cerebral edema, Guillain–Barre syndrome, benign intracranial hypertension, meningoencephalitis, and acute disseminated encephalomyelitis (20, 50, 52). Cerebral venous thrombosis, in particular, is closely associated with COVID-19, and its underlying mechanisms include immune system dysregulation, cytokine storms, increased blood viscosity, thrombogenesis, hypercoagulability, and inflammation (49, 53).

Fever is also the most common sign in KD. It is usually higher than 39°C, and it can be persistent for approximately 3 weeks without treatment. Conjunctival injection can be present, bilateral, and non-purulent. A careful history may indicate the presence of signs and symptoms like conjunctival injection before the hospital presentation. Other important signs include cracking of the lips, erythema, a strawberry-colored tongue, and oral ulceration, none of which can definitively diagnose KD. The lymphadenopathy, if present, is usually unilateral. The rash onset occurs 5 days after the fever, and it can be polymorphous, usually being maculopapular or scarlatiniform. It is usually expressed on the trunk and extremities and can undergo desquamation in its evolution (54).

Patients with MIS-C commonly present with prominent cardiac involvement manifested by left ventricular (LV) systolic and diastolic dysfunction, myocardial inflammation, and coronary artery dilations. LV dysfunction is more severe in patients with MIS-C than in patients with KD (48). Although ventricular dysfunction and CAAs are the predominant risk factors for morbidity in patients with multisystemic inflammatory syndrome, the mechanism is not completely known (33). Some clinical studies have shown the presence of the SARS-CoV-2 virus in the heart, lungs, and kidneys of patients with severe cardiac involvement with or without underlying conditions prior to the viral infection (55, 56). In contrast with MIS-C patients, who generally experience fewer coronary artery abnormalities that are usually transient and resolve quickly, KD primarily affects the coronary arteries, resulting in dilation and aneurysms (as seen in **Table 1**). While small to moderate CAAs in KD may take up to 2 years to normalize, the rapid resolution of cardiovascular complications in MIS-C after the acute phase suggests that these issues are likely due to hyperinflammation causing capillary leakage and vasodilation, rather than direct immune cell damage to the myocardium (57, 58). KD can also lead to myocarditis, which is associated with arrhythmias and potential long-term fibrosis (59).

**Table 1:** Clinical comparison between MIS-C and Kawasaki disease (3, 13, 22, 23, 50–53).

Comparison	MIS-C	Kawasaki disease
Age	6–12 years	6 months to 5 years
Sex	Male predominance	Male predominance
Race or ethnicity	African, Hispanic, and South Asian	Japanese and Asian
Similarities	Fever, rash, cervical lymphadenopathy, neurological symptoms, and extremity changes	
Differences	Relatively high incidence of gastrointestinal symptoms, myocarditis, shock, and coagulopathy	Relatively high incidence of conjunctival injection and oral mucous membrane changes
Cardiovascular involvement	Left ventricle dysfunction	Coronary aneurysm

MIS-C, multisystem inflammatory syndrome in children.

## 5 Diagnosis

The differences from the 2020 CDC case definition for MIS-C include the removal of the fever duration requirement and the specification of systemic inflammation as indicated by a C-reactive protein level of ≥3.0 mg/dL. Additionally, respiratory, renal, and neurologic systems are no longer included in the organ involvement criteria, while shock has been added as a distinct organ system manifestation. SARS-CoV-2 testing criteria now include time parameters, such as viral testing within 60 days of MIS-C hospitalization or serologic testing during the illness (32). The diagnosis criteria encompass clinical signs and symptoms, laboratory tests, and epidemiologic linkage, as detailed in **Tables 2**–**4**.

**Table 2:** MIS-C—clinical diagnosis criteria [adapted from (60)].

No.	Clinical criteria An illness in a person aged <21 characterized by all of the following, in the absence of a more likely alternative diagnosis				
1	Fever: temperature ≥ 38.0°C. It can be subjective or documented.			
2	Clinical severity: requiring hospitalization or resulting in death.				
3	Systemic inflammation: indicated by C-reactive protein with values ≥3.0 mg/dL (30 mg/L).				
4	New onset manifestations in at least two of the following categories:				
	Cardiac involvement indicated by one of the following: left ventricular ejection fraction <55%, coronary artery dilatation, aneurysm, or ectasia, troponin elevated above laboratory normal range.	Mucocutaneous involvement indicated by rash, inflammation of the oral mucosa (e.g., mucosal erythema or swelling, drying or fissuring of the lips, and strawberry tongue), conjunctivitis or conjunctival injection (redness of the eyes), and extremity findings (e.g., erythema or edema of the hands or feet).	Shock: clinical finding.	Gastrointestinal involvement is indicated by abdominal pain, vomiting, and diarrhea.	Hematologic involvement indicated by platelet count <150,000 cells/μL or lymphocyte count <1,000 cells/μL.

MIS-C, multisystem inflammatory syndrome in children.

**Table 3:** MIS-C—laboratory diagnosis criteria (adapted from (60)).

No.	Laboratory criteria
1	Detection of SARS-CoV-2 ribonucleic acid (RNA) up to 60 days prior to or during hospitalization, or in a post-mortem specimen using a diagnostic molecular amplification test [e.g., polymerase chain reaction (PCR)] or
2	Detection of SARS-CoV-2-specific antigen up to 60 days prior to or during hospitalization, or in a post-mortem specimen, or
3	Detection of SARS-CoV-2-specific antibodies in serum, plasma, or whole blood associated with current illness resulting in or during hospitalization.
Requires a positive serology test regardless of COVID-19 vaccination status. Detection of anti-nucleocapsid antibodies is indicative of SARS-CoV-2 infection, while anti-spike protein antibodies may be induced either by COVID-19 vaccination or by SARS-CoV-2 infection.	

MIS-C, multisystem inflammatory syndrome in children.

**Table 4:** MIS-C—epidemiologic linkage [adapted from (60)].

No.	Epidemiologic linkage
1	Close contact with a confirmed or probable case of COVID-19 disease in the 60 days prior to hospitalization.
2	Close contact is generally defined as being within 6 feet for at least 15 minutes (cumulative over a 24-hour period). However, it depends on the exposure level and setting; for example, in the setting of an aerosol-generating procedure in healthcare settings without proper personal protective equipment (PPE), this may be defined as any duration.

MIS-C, multisystem inflammatory syndrome in children.

### 5.1 Other criteria

#### 5.1.1 Vital record criteria

A person should be aged <21 years whose death certificate lists MIS-C or multisystem inflammatory syndrome as an underlying cause of death or a significant condition contributing to death.

### 5.2 Criteria to distinguish a new case from an existing case

A case should be recorded as new if the person has never been previously recorded as a case, or if the most recent case was recorded with an illness onset date (if available) or a hospital admission date more than 90 days prior. A patient is suspected of having MIS-C when the vital criteria are present. A case is confirmed if it meets both the clinical and confirmatory laboratory criteria. A diagnosis of MIS-C is considered probable if the clinical criteria and epidemiologic linkage are positive (60).

As for KD, there are two types of disease, known as complete KD and incomplete KD, further discussed in **Tables 5**, **6**.

**Table 5:** Complete Kawasaki disease—diagnostic criteria (45, 46, 51).

No.	Complete Kawasaki disease Fever persisting for at least 5 days + at least 4 of the following 5 criteria:
1	Bulbar conjunctival infection. It is bilateral, non-exudative, and painless.
2	Rash: often maculopapular, erythema multiforme, or scarlatiniform. It occurs in the first few days, and it involves the trunk and the extremities.
3	Lips: erythema and cracking of the lips and strawberry tongue. It can involve all the oral and pharyngeal mucosa, and it is non-exudative.
4	Edema: it affects the hands and feet, and it can be associated with erythema. It is painful, and it is followed by desquamation in the second week of illness.
5	Lymphadenopathy: cervical, unilateral, and bigger than 1.5 cm in diameter.
If more than 4 principal clinical criteria are present, the diagnosis can be established earlier with only 4 days of fever. If coronary artery abnormalities are present, they can be diagnosed by less than four of the criteria mentioned.	

**Table 6:** Incomplete Kawasaki disease—diagnostic criteria (48, 49).

No.	Incomplete Kawasaki disease
1	Occurs in patients with fever for more than 5 days but with 2 or 3 of the clinical findings criteria (conjunctival infection, rash, lips erythema, extremities edema, lymphadenopathy). Abnormal investigation results are required to support the diagnosis (such as platelet count >450000/mm3).
2	The patients with an incomplete Kawasaki disease picture have a higher risk of cardiac complications.
3	Other findings in addition to diagnostic criteria are: neurological such as aseptic meningitis, abdominal pain, vomiting, diarrhea, arthralgia, arthritis, and dysuria.
4	The inflammation of BCG site it’s a common finding in Kawasaki disease and MIS-C.

MIS-C, multisystem inflammatory syndrome in children.

## 6 Investigations

Lymphopenia is a characteristic of COVID-19 and is more pronounced in MIS-C cases compared to children with mild SARS-CoV-2 infection or KD (34). Another significant observation is the low platelet count, which is a feature of MIS-C, in contrast to KD where thrombocytosis is more common. However, a decline in platelet count is a frequent element of TSS, which was described in 5% of the cases diagnosed with KD (61).

Regarding MIS-C, C-reactive protein, erythrocyte sedimentation rate, and ferritin levels may also be elevated. Abnormal coagulation parameters, including increased D-dimer, fibrinogen, and international normalized ratio, have been observed (62). Additionally, elevated levels of N-terminal pro-BNP and troponin have been reported to suggest myocardial damage (62–64). Since patients with KD typically exhibit lower inflammatory markers, it is suggested that inflammation plays a crucial role in the more severe cardiovascular complications seen in MIS-C (61).

In KD, the leukocyte count is characterized by neutrophilia and lymphopenia with elevated levels of C-reactive protein and erythrocyte sedimentation rate, thrombocytosis, and elevated levels of alanine aminotransferase and aspartate aminotransferase. Decreased levels of albumin, hemoglobin, sodium, potassium, and cholesterol have also been detected (64).

In comparison, studies have shown that MIS-C patients tend to have lower levels of leukocytes, lymphocytes, and platelets but higher levels of C-reactive protein, D-dimer, and ferritin. Meanwhile, patients with MIS-C appear to have similar levels of procalcitonin and erythrocyte sedimentation rate compared to KD. Concerning the cardiac markers, MIS-C patients had higher levels of creatine phosphokinase (CPK), and no significant differences were found as regards N-terminal pro-BNP levels or troponin levels. MIS-C patients can have lower levels of albumin, sodium, and potassium, while KD patients can have higher levels of creatinine (65, 66). A detailed list of investigations conducted for the diagnosis of MIS-C and KD is presented in **Table 7**.

**Table 7:** Paraclinical investigations in MIS-C and Kawasaki disease (67–70).

MIS-C—paraclinical investigations	For all patients consider	First-line tests	• A nasopharyngeal COVID-19 polymerase chain reaction (PCR) test on presentation. • COVID-19 serology. • A complete blood count and C-reactive protein on presentation, erythrocyte sedimentation rate. • Renal and liver function tests.
		Second-line tests	• Ferritin as a second-line investigation. • Coagulation panel, fibrinogen, and D-dimers. • Troponin levels and brain natriuretic peptide/pro-brain natriuretic peptide should also be considered. • Blood culture, urine culture, and respiratory pathogen panel. • Electrocardiogram. • Echocardiography. • Chest radiographs. • Abdominal echography. • Cytokine panel. • Viral serologies and blood PCR for pathogen-specific infections. • Antiphospholipid panels. • Complement workup. • Quantitative serum immunoglobulins.
Kawasaki disease—paraclinical investigations	There is no diagnostic test for KD.	• Blood tests: complete blood count in the acute stage often finds mild to moderate normochromic anemia, and in the subacute stage, it is common to find thrombocytosis, CRP, ESR, renal and liver function, albumin level, blood culture, Antistreptolysin O Titer (ASOT), urinalysis (sterile pyuria can also be present due to urethral inflammation), and culture, COVID-19 swab. • Echocardiogram: at presentations, 2 and 6 weeks after the initial tests. • Electrocardiogram. • Urine meprin A or filamin C (as a marker of Kawasaki disease activity).	

MIS-C, multisystem inflammatory syndrome in children; CRP, C-reactive protein; ESR, erythrocyte sedimentation rate.

## 7 Treatment

### 7.1 MIS-C

The initial treatment includes both immunomodulatory and antithrombotic therapy. Immunomodulatory medications intravenous immunoglobulin (IVIG) and glucocorticoids are the most common immunomodulatory medications used for children diagnosed with MIS-C. It is postulated that the association of these two therapeutic methods decreases the treatment failure rate and results in enhanced cardiac function recovery with shorter intensive care unit stays (2).

Anakinra, a recombinant IL-1 receptor antagonist, is a well-established treatment for MAS and is increasingly used in children with MIS-C who have refractory disease unresponsive to glucocorticoid therapy or when steroids are contraindicated. Its main advantages include a short half-life and a rapid onset of action (71, 72).

Infliximab is a chimeric monoclonal antibody that binds to TNF-α and inhibits its downstream pro-inflammatory effects. It is used to treat IVIG refractory MIS-C and KD (57). Tocilizumab is a recombinant humanized anti-IL6R monoclonal antibody, which is not recommended for all pediatric patients due to the lack of benefit in randomized studies, the risk of infections, and the long half-life (73, 74).

Antithrombotic therapy includes the use of antiplatelets such as aspirin in low doses for all patients without risk factors for bleeding. In addition, anticoagulants are used for patients with large coronary aneurysms and also for those who have moderate to severe LV dysfunction but no risk for bleeding. For all MIS-C patients without aneurysms or cardiac dysfunction, prophylactic or therapeutic anticoagulation should be considered on an individual basis. This decision should take into account risk factors for thrombosis and bleeding, including malignancy, critical illness, obesity, pre-existing inflammatory disease, history of thrombosis, inherited thrombophilia, immobility, and indwelling central lines (75).

The recommended aspirin dosage is 3–5 mg/kg/day orally, once daily, with a maximum dose of 81 mg/day. This dosage should be avoided in cases when the platelet count is <80,000, fibrinogen is <100 mg/dL, or if there is active bleeding or a high risk of bleeding (76–78). Aspirin may be combined with a prophylactic dose of enoxaparin for coronary artery protection (79, 80).

Anticoagulant therapy should be considered in patients presenting with any of the following: acute thrombosis, moderate to severe ventricular dysfunction, coronary dilatation/aneurysm with a Z-score > 10, or D-dimer levels 10 times the upper limit of normal. Unfractionated heparin, direct thrombin inhibitors, and low-molecular-weight heparin can be used, with anti-factor Xa activity monitored. Prophylactic anticoagulant therapy should also be considered alongside aspirin in patients with one of the following conditions: venous thromboembolism, mild to moderate ventricular dysfunction, coronary dilatation/aneurysm with a Z-score of 2.5–10, or D-dimer levels 5–10 times the upper normal limit. The recommended enoxaparin dose is 0.5 mg/kg every 12 hours, with a maximum dose of 30 mg for patients aged >2 months to <18 years (81).

Post-discharge anticoagulation: Patients with MIS-C and documented thrombosis or an ejection fraction >35% should receive therapeutic anticoagulation for at least 2 weeks after discharge from the hospital (71).

In conclusion, enoxaparin is widely used for thromboprophylaxis in MIS-C due to its role in inhibiting thrombin and factor Xa, thereby reducing fibrin clot formation and preventing thromboembolic events, which are often seen in MIS-C due to endothelial dysfunction and hypercoagulability (82). However, cytokine inhibitors such as anakinra, an IL-1 receptor antagonist, and tocilizumab, an IL-6 receptor antagonist, specifically target the pro-inflammatory cytokines, driving the hyperinflammatory response in MIS-C (72). By blocking these inflammatory signals, these inhibitors help modulate the immune system and mitigate the tissue damage and organ dysfunction associated with the condition.

Other therapies, including extracorporeal membrane oxygenation (ECMO), should be considered in cases of cardiorespiratory failure refractory to treatment (83). Therapeutic plasma exchange has been used in critically ill patients who have not responded to third-line immunomodulatory treatment. It is not routinely used for patients with MIS-C (84).

### 7.2 Kawasaki disease treatment

The acute phase treatment starts with IVIG (85, 86). The mechanism of action of IVIG in the treatment of KD is unknown, but it may include the modulation of cytokine production, neutralization of toxins, augmentation of regulatory T-cell activity, suppression of antibody synthesis, and provision of anti-idiotypic antibodies (87). IVIG is administered at a dosage of 2 g/kg, given over 12 hours, along with oral aspirin. Aspirin, during the acute phase of illness, should be administered at high or moderate doses every 6 to 8 hours (88, 89). Low-molecular-weight heparin should be given to patients with large aneurysms and with a high risk for thrombosis and rupture. It is recommended to be associated with aspirin. Warfarin is preferred in the acute phase due to its anti-inflammatory effect and remodeling action (89, 90).

### 7.3 Refractory Kawasaki disease

Several studies have suggested trying a second IVIG infusion for patients with fever, but no treatment guidelines are available (91). Infliximab is noted to reduce fever and both the duration of hospitalization and the incidence of hospital admissions, although no effect has been observed on coronary artery outcomes (92). Etanercept, a biological TNF inhibitor, acts as a soluble TNF receptor that binds TNF-α. It has been shown to reduce fever and inflammation (93). Atorvastatin, known for its antioxidant effects, has been concluded to be safe for use in young children with periodic monitoring (94). Cyclosporine has been studied in patients with refractory KD, and a recent study in Japan has completed enrollment (95, 96).

Scoring systems have been developed to predict resistance to IVIG treatment. One such system assigns 1 point for infants younger than 6 months, before 4 days of illness, platelet counts higher than 300,000 per mm^3^, and C-reactive protein levels higher than 8 mg/dL. An additional 2 points are assigned for Alanine Aminotransferase (ALT) levels equal to or higher than 80 IU/L. This scoring system demonstrated a sensitivity of 78% and a specificity of 76%, with a cutoff score of 3 or more to indicate IVIG resistance (97).

The treatment doses are outlined in **Table 8**.

**Table 8:** MIS-C and Kawasaki disease treatment comparison (57, 71–81, 85–96).

	MIS-C	Kawasaki disease	
		*Responsive Kawasaki* *disease*	*Refractory Kawasaki* *disease*
Intravenous immunoglobulin	2 mg/kg based on ideal body weight	2 g/kg every 12 hours along with oral aspirin	2 g/kg/day
Glucocorticoids	1–2 mg/kg/day methylprednisolone or IV pulse 10–30 mg/kg/day		
Anakinra	>4 mg/kg/day		
Infliximab	5–10 mg/kg IV for 1 dose		5 mg/kg single-dose infusion
Aspirin	3–5 mg/kg/day orally once daily to a maximum dose of 81 mg/day unless the platelet count is <80,000, fibrinogen < 100 mg/dL, active bleeding, or high risk of bleeding	30–100 mg/kg/day High dose: 50–100 mg/kg/day	
Enoxaparin	1 mg/kg Subcutaneous (SUBQ) every 12 hours (up to a maximum of 30 mg for ages >2 months and <18 years)	1 mg/kg/dose subcutaneously every 12 h	
Etanercept			0.4–0.8 mg/kg subcutaneous weekly for 3 doses

MIS-C, multisystem inflammatory syndrome in children.

## 8 Outcome

Although most cases of MIS-C require intensive care unit admission, studies have shown that only one-third of these cases require mechanical ventilation, and an even smaller number need ECMO (98, 99). The majority of patients recover, but cases of death are also reported. In 2024, 117 cases of MIS-C with illness onset in 2023 were reported in the United States. Among these cases, the median age was 7 years, and 68% had no underlying medical conditions. Of these patients, 50% needed intensive care, 34% experienced shock, and 29% had cardiac dysfunction. The mortality rate was 3%. Although 96% of patients were eligible for vaccination, only 18% had documented vaccination, and 60% of them had received their last dose more than 12 months before MIS-C onset (66). A 2021 study conducted over 6 months in the United States examined 102 MIS-C cases in patients aged 12–18 years. It found that only 5% of patients were fully vaccinated with two doses at least 28 days before hospitalization, while 95% were unvaccinated. Notably, all 38 MIS-C patients who required life support were unvaccinated. No deaths were reported in the study, and the length of hospital stay was similar between vaccinated and unvaccinated patients (100).

As for KD, early detection and treatment usually lead to complete recovery approximately 6 weeks after the onset of symptoms (101). In the acute stage, immunoglobulins administered at high doses may decrease coronary artery injury, but 15%–20% of such cases will develop IVIG resistance (90). According to the literature, the incidence of CAA is nine times higher in cases of KD that are resistant to IVIG treatment compared to those that are IVIG-sensitive (91, 102).

## 9 Discussions

While both MIS-C and KD share several clinical features, they are distinct entities with important differences in their origins and overall impact. MIS-C, a post-infectious hyperinflammatory syndrome, has been closely linked to SARS-CoV-2 infection and typically affects older children and adolescents (1). KD, by contrast, has a longer-established history, generally affecting younger children under the age of 5, with its precise cause still unclear (9, 12). It is believed to arise from a combination of genetic and environmental factors, yet no specific infectious agent has been identified, unlike the clear viral association seen with MIS-C during the COVID-19 pandemic (25, 26, 103).

The inflammatory mechanisms behind each condition, while similar in triggering systemic inflammation, lead to different patterns of organ involvement. MIS-C is characterized by a heightened and more severe immune response, often involving multiple organs, including the heart, gastrointestinal tract, and central nervous system (32, 33). This extensive involvement leads to more severe presentations, including shock and myocardial dysfunction. KD, while also inflammatory in nature, tends to focus more narrowly on the coronary arteries, resulting in the development of CAAs if left untreated. While both conditions are driven by an overactive immune system, the severity of multisystem involvement in MIS-C often presents a more critical situation compared to KD, where coronary artery complications are the primary concern (37).

In terms of clinical presentation, both syndromes can present with common symptoms such as persistent fever, rash, conjunctivitis, and mucosal inflammation, which can make differentiation challenging, especially in the early stages. However, MIS-C is more likely to involve significant gastrointestinal symptoms, such as abdominal pain, vomiting, and diarrhea, as well as neurological signs like confusion and headaches (48). Cardiovascular involvement is also more pronounced in MIS-C, with many patients developing hypotension or shock (49). KD, however, typically follows a more predictable course, presenting with prolonged fever, mucocutaneous signs, and swelling of the extremities. Coronary artery changes are specific to KD and serve as a critical differentiating factor, although they tend to develop later in the disease process (57, 59). Laboratory findings provide further clues that help distinguish between these two conditions. In MIS-C, inflammatory markers such as C-reactive protein, ferritin, and D-dimer levels are often markedly elevated, and there is frequent evidence of cardiac injury, such as elevated troponin or BNP. Lymphopenia and thrombocytopenia are also more common in MIS-C. In contrast, KD is often characterized by elevated inflammatory markers as well, although thrombocytosis may be seen in later stages (65, 66). Evidence of SARS-CoV-2 exposure or infection is a key diagnostic factor for MIS-C, while the diagnosis of KD relies on clinical criteria, with no identifiable infectious trigger.

Despite these differences, treatment approaches overlap, with IVIG serving as a primary therapy for both conditions (76). In MIS-C, however, the more severe inflammatory response often necessitates the use of additional therapies such as corticosteroids or biologics like anakinra or infliximab (57, 71). Patients with MIS-C may require intensive care due to cardiovascular involvement and shock, whereas KD patients generally respond well to IVIG and aspirin, which helps prevent coronary artery complications (76). The aggressive nature of MIS-C requires close monitoring and more intensive treatment to prevent serious complications such as multiorgan failure. Ultimately, both conditions, when recognized and treated early, have favorable outcomes. However, the potential for severe complications is higher in MIS-C due to its rapid progression and widespread organ involvement. Ongoing research will continue to clarify the pathophysiological mechanisms and treatment strategies for these complex syndromes, ensuring better care for affected children and improving long-term outcomes (99).

## 10 Conclusion

MIS-C and KD are distinct inflammatory conditions with overlapping features but different causes and treatment approaches. MIS-C, a post-viral syndrome associated with SARS-CoV-2, triggers widespread systemic inflammation and can lead to severe complications, particularly affecting the cardiovascular system. It is thought to result from an exaggerated immune response, requiring timely intervention with IVIG, corticosteroids, and sometimes biological therapies to manage inflammation and prevent organ failure.

KD, primarily affecting young children, remains a leading cause of coronary artery abnormalities. Although its exact cause is unknown, genetic and environmental factors are believed to play roles. Left untreated, it can result in CAAs, making IVIG and aspirin essential in reducing inflammation and preventing long-term cardiovascular damage.

Both conditions show elevated inflammatory markers and share some treatment strategies, but MIS-C tends to cause more extensive organ involvement, requiring prompt medical intervention. Accurate diagnosis and early treatment are crucial in preventing complications. As research progresses, a deeper understanding of these syndromes will improve patient outcomes and refine treatment strategies tailored to their distinct pathophysiological mechanisms.
